# Does surgeon expertise influence long-term outcomes in ovarian endometrioma rupture cases?

**DOI:** 10.1186/s12905-026-04606-9

**Published:** 2026-06-24

**Authors:** Shiqing Lyu, Yushi Wu, Yi Dai, Xiaoyan Li, Jinghua Shi, Zhiyue Gu, Chenyu Zhang, Qiutong Li, Hailan Yan, Biyun Zhang, Jinhua Leng

**Affiliations:** 1 Department of Obstetrics and Gynecology, Peking Union Medical College Hospital, Chinese Academy of Medical Sciences & Peking Union Medical College, No. 1 Shuaifuyuan, Dongcheng District, Beijing, 100730 China; 2 National Clinical Research Center for Women’s Health and Obstetric and Gynecologic Diseases, Beijing, China

**Keywords:** Cystectomy, Endometriosis, Expertise, Surgery, Recurrence

## Abstract

**Background:**

Spontaneous ovarian endometrioma rupture typically presents with acute abdominal pain, a condition often complicated by tissue edema and pelvic adhesions that increase surgical difficulty.

This study aims to evaluate the impact of surgical expertise on long-term outcomes in patients with a history of spontaneous ovarian endometrioma rupture.

**Methods:**

This is a retrospective cohort study at Peking Union Medical College Hospital between January 2012 and December 2022, which analyzed patients with spontaneous ovarian endometrioma rupture who underwent surgery. Patients were categorized into specialist or non-specialist surgery groups based on the expertise level of the surgical team. Clinical characteristics, postoperative treatment and recurrence data were collected and compared. Of the 122 patients, 32 were treated by specialists and 90 by non-specialists. All participants received laparoscopic surgery.

**Results:**

Baseline characteristics and intraoperative findings were comparable between the two groups. Elective surgery was performed more frequently in the specialist group (81.2%) than in the non‑specialist group (45.6%, p = 0.001). The crude recurrence risk was 15.6% (5/32) in the specialist group, compared with 33.3% (30/90) in the non-specialist group (p = 0.264). Time-to-event analysis demonstrated a significantly lower 10-year cumulative recurrence risk in the specialist group (log-rank p = 0.033). In multivariable Cox regression analysis adjusting for surgery timing, maximum ovarian endometrioma diameter, rASRM score, and treatment duration, specialist surgery remained associated with a lower hazard of recurrence (adjusted HR 0.378, 95% CI 0.140–1.021), although this did not reach statistical significance (p = 0.055). None of the other covariates were significantly associated with recurrence. No between‑group difference was observed in clinical pregnancy rates.

**Conclusions:**

For patients with a history of spontaneous rupture of ovarian endometrioma, surgery performed by specialists with more extensive experience in endometriosis may be associated with a lower long-term recurrence risk. This finding should be interpreted with caution given the borderline statistical significance after Cox adjustment. Nonetheless, the observed trend highlights the potential importance of surgical expertise and the need for specialized training in endometriosis management.

## Background

Ovarian endometriosis, also known as ovarian endometrioma (OMA), represents a significant subset of endometriosis, accounting for 17–44% of all cases [1]. It is marked by the formation of cysts from ectopic endometrial tissue, which closely resembles the eutopic endometrium both histologically and functionally. Patients with OMA may experience various clinical symptoms, including pain and infertility.

During menstruation, cyst enlargement due to increased intracystic pressure may result in spontaneous rupture. The leakage of chocolate-colored contents may trigger chemical peritonitis and acute abdominal pain, a frequent reason for emergency gynecological visits [2]. Our earlier study reported a 9% spontaneous rupture rate—higher than previously documented [3]. The ensuing inflammatory response may exacerbate pelvic adhesions, complicating subsequent surgery.

Laparoscopic cystectomy is advocated as a treatment for endometriosis-associated symptoms [4], but recurrence remains common after primary surgery (30.0–50.0% at five years) [5]. While complete lesion removal can reduce recurrence, it may also damage healthy ovarian tissue and impair fertility. This highlights the need to balance surgical thoroughness with ovarian preservation, particularly in reproductive-aged women.

Surgeons with extensive experience in endometriosis-specific procedures and management are likely to achieve better fertility outcomes compared with general gynecologists lacking specialized training [6]. However, the impact of surgical expertise on outcomes following surgery for ruptured OMAs—particularly regarding recurrence—remains underexplored.

This retrospective study aims to evaluate the long-term impact of surgical expertise on recurrence risk in patients with a history of ruptured OMA, comparing surgeries performed by a specialist in endometriosis versus general gynecologists.

## Methods

**Fig. 1:**
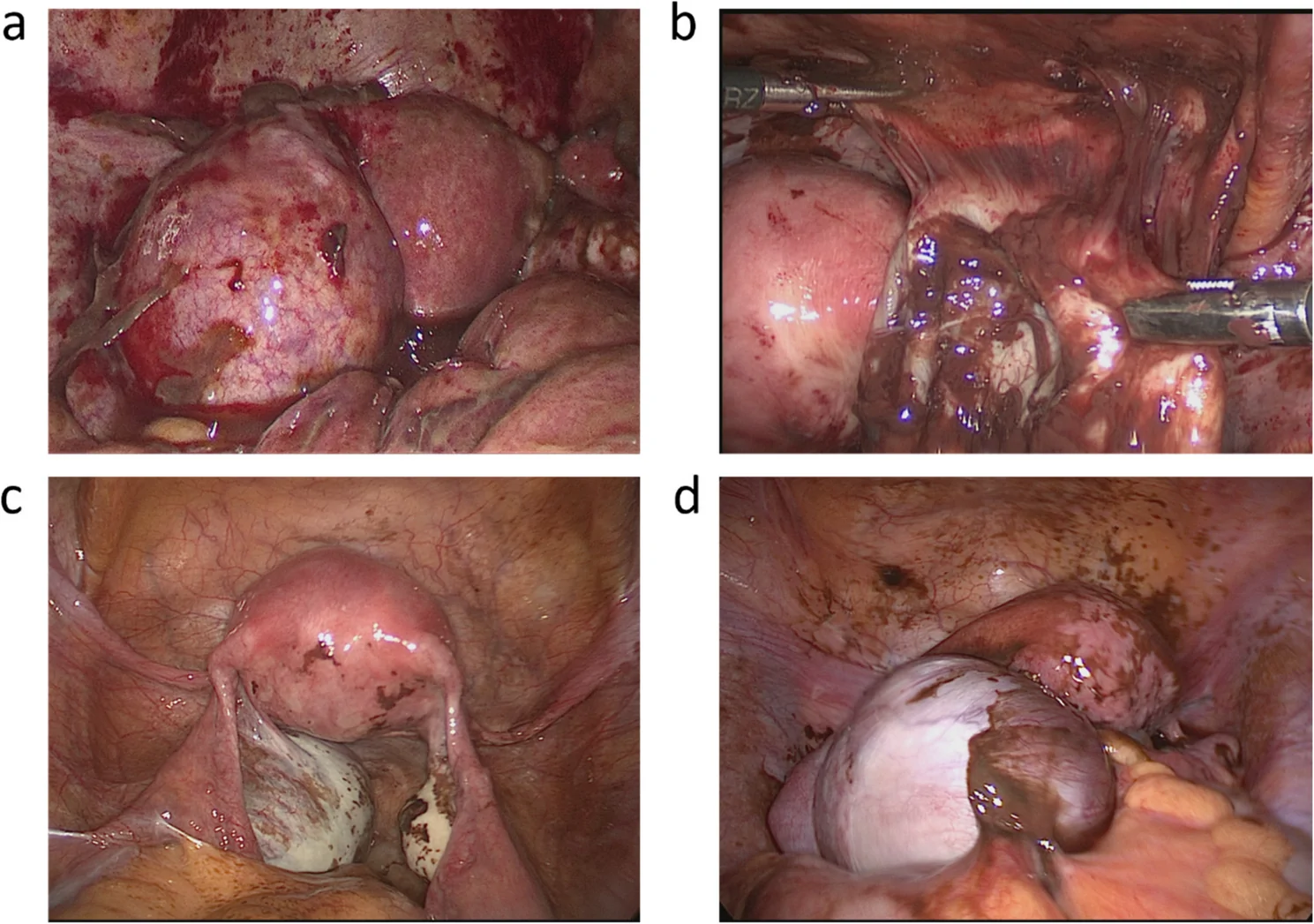
Signs of ruptured OMA during surgery. **a**, **b **Cases of OMA rupture emergency. Chocolate-like staining is distributed on the surface of pelvic organs. Tissue edema and adhesion can be seen. **c**, **d** Signs of ruptured OMA in elective surgery. Dark brown plaques are found on the pelvic organs and visceral peritoneum

This retrospective study analyzed spontaneous OMA rupture cases treated surgically at Peking Union Medical College Hospital (PUMCH) from January 2012 to December 2022. Eligible participants were women aged 20 to 40 years with histopathologically confirmed OMA diagnoses. Cases associated with borderline ovarian tumors or reproductive malignancies were excluded. All included patients presented with acute abdominal pain and were subsequently diagnosed with ruptured OMA based on intraoperative findings during laparoscopic cystectomy. Intraoperative signs of rupture included chocolate-like staining on the peritoneal surface, omentum, or pelvic organs (Fig. 1). The study was approved by the Ethics Committee of PUMCH (I-23PJ2095) in accordance with the Declaration of Helsinki.

Participants were stratified according to the experience level of the surgeon in endometriosis management. The specialist group consisted of patients operated on by gynecologists from the hospital’s Center for Endometriosis, each with over ten years of dedicated experience in performing endometriosis-related surgeries, including procedures for OMA and deep infiltrating endometriosis. The non-specialist group included more than 20 gynecologists, each with less than ten years of relevant experience in endometriosis or without subspecialty focus. In contrast, the specialist group comprised four surgeons, all of whom are members of the *Committee of Endometriosis*,* Obstetrics and Gynecology Branch*,* Chinese Medical Doctor Association*—a nationally recognized expert group specializing in endometriosis care. Laparoscopic cystectomy was performed in all cases and consisted of cyst wall stripping, adhesion lysis, and hemostasis. The surgical principle was consistent across patients, although specific instruments and energy modalities varied according to surgeon preference.

Data collected for each patient included general demographic characteristics (age and parity) as well as surgical findings, such as cyst size and laterality, presence of adhesions, deep infiltrating endometriosis, cul-de-sac obliteration, and method of hemostasis. Follow-up information on postoperative treatment, recurrence, and fertility outcomes was obtained through outpatient clinic visits or standardized phone interviews. Cyst recurrence was defined as the presence of an ovarian cyst with a thin wall, measuring at least 2 cm in diameter, containing homogenous low echogenic fluid with scattered internal echoes, and persistence across menstrual cycles. Pain recurrence was considered present if symptoms such as dysmenorrhea, dyspareunia, or pelvic pain reappeared three months or more after surgery. Clinical pregnancy was defined as a pregnancy lasting ≥ 12 weeks of amenorrhea with ultrasonographic confirmation of an intrauterine gestational sac. All patients underwent surgery at different time points over a ten-year period. Follow-up was conducted retrospectively until the end of the study period, capturing either the time from surgery to recurrence or to the last follow-up if no recurrence was observed. For patients who had not experienced recurrence by the end of follow-up, their data were censored accordingly.

The study aimed to compare the recurrence risk between the specialist and non-specialist surgical group. A stratified analysis was performed according to the clinical context at the time of surgery (emergency versus elective), in order to explore whether the association between surgical expertise and outcomes was consistent across different clinical scenarios. Emergency surgery was defined as urgent surgical intervention undertaken due to acute symptoms following ovarian endometrioma rupture, at the discretion of the attending physician. Elective surgery referred to planned laparoscopic cystectomy performed after initial conservative management and clinical stabilization, usually scheduled several months after initial symptoms resolution.

Statistical analyses were conducted using IBM SPSS Version 25.0 (IBM Corp., Armonk, NY, USA). Continuous variables were presented as mean with standard deviation or median with interquartile range, and categorical variables as percentages. Quantitative variables were compared using the Student’s t-test or Mann-Whitney U test, and qualitative variables were compared using the chi-square or Fisher’s exact test, as appropriate. The ten-year cumulative hazard of recurrence between the two groups was estimated using Kaplan-Meier survival analysis. To further evaluate factors associated with recurrence, multivariable Cox proportional hazards regression analyses were performed, with results presented as hazard ratios (HRs) and 95% confidence intervals (CIs). Variables included in the multivariable model were selected based on clinical relevance, including surgery timing, OMA diameter, revised American Society of Reproductive Medicine (rASRM) score, and treatment duration. All statistical tests were two-tailed, and a P value < 0.05 was considered statistically significant.

## Results

**Fig. 2:**
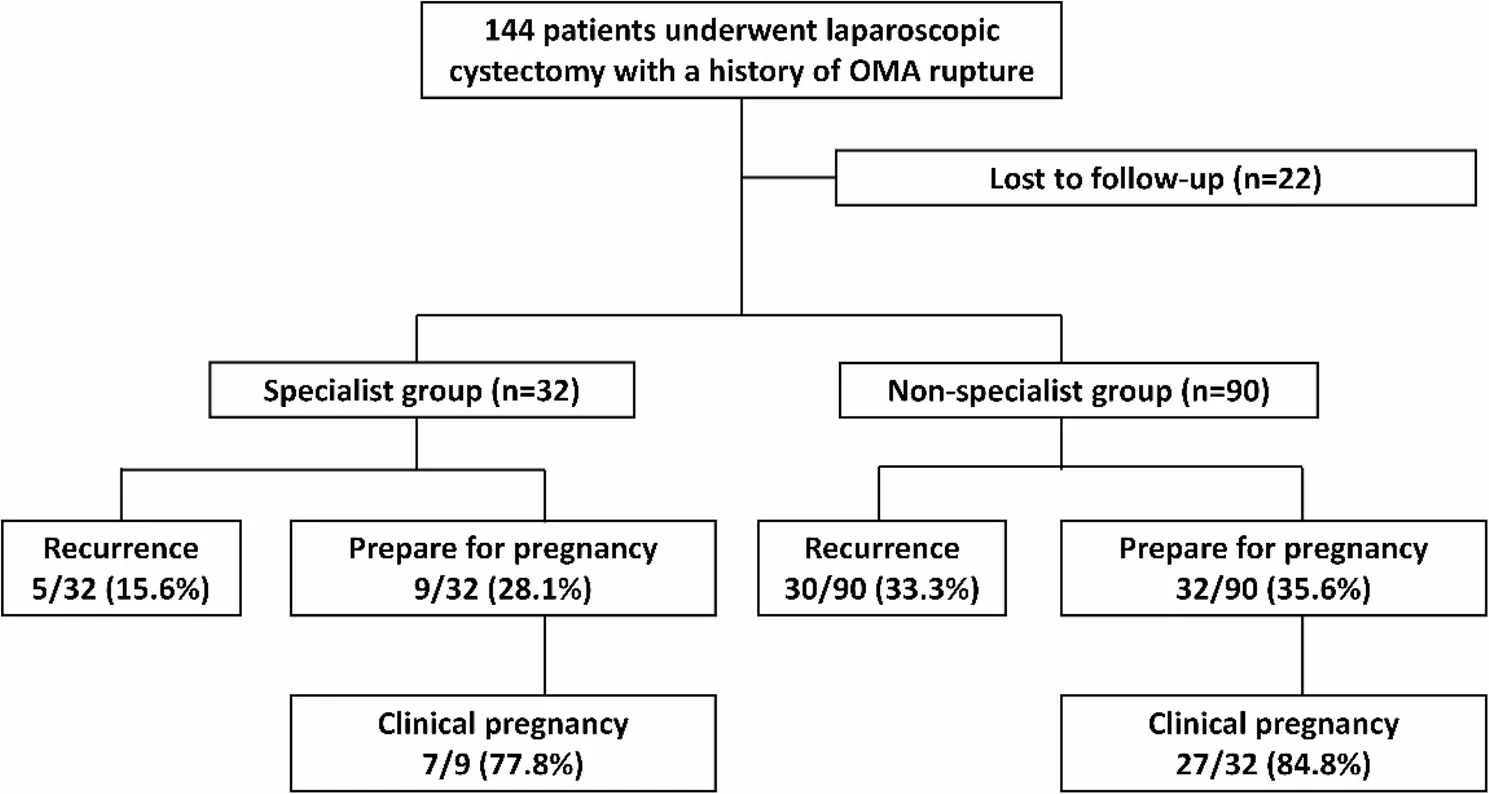
The flow diagram of included patients

A total of 122 patients were included in the study, with 32 in the specialist group and 90 in the non-specialist group (Fig. 2). Baseline demographic characteristics, including age, reproductive history, symptoms of dysmenorrhea, preoperative serum CA125 levels, and OMA diameter, were comparable between the two groups. Notably, a significantly higher percentage of patients in the specialist group (81.2%) underwent elective surgery, compared with 45.6% in the non-specialist group (*p* = 0.001).

Regarding intraoperative information, no significant differences were observed between the two groups in terms of bilateral OMA incidence, presence of pelvic adhesions, deep infiltrating endometriosis, cul-de-sac obliteration, rASRM score, or coexisting adenomyosis. Specialists appeared to more frequently utilize coagulation for hemostasis, but no statistically significant difference was observed. Surgeries performed by specialists were associated with shorter operative time and reduced intraoperative blood loss in median values; however, these differences also did not reach statistical significance.

Postoperative treatment was administered to 75.0% of patients in the specialist group and 66.7% in the non-specialist group, with no significant difference. Treatment modalities included gonadotropin-releasing hormone agonists, oral contraceptives, dienogest, intrauterine devices, or combinations. There were no statistically significant differences between the two groups in terms of treatment regimens or overall postoperative treatment rates. The median duration of postoperative hormonal therapy was 5.00 months [1.00, 19.00] in the specialist group and 3.00 months [1.50, 7.25] in the non-specialist group, with no statistically significant difference (*p* = 0.284). The proportion of patients receiving long-term therapy (> 1 year) was higher in the specialist group (38.7% vs. 21.7%), although this difference did not reach statistical significance (*p* = 0.110).

Among all patients with follow-up data in the specialist group, 15.6% (5/32) experienced recurrence, with no recurrence within the first six months. In comparison, the non-specialist group showed a higher crude recurrence risk of 33.3% (30/90), although this difference did not reach statistical significance (*p* = 0.264). Notably, 30% (9/30) of these recurrences occurred within the first six months (*p* = 0.385) (Table 1). At five years, the cumulative recurrence risk was 34.5% in the non-specialist group and 26.9% in the specialist group. Stratified analysis by surgical context showed a statistically lower recurrence risk in the specialist group among elective cases, while no clear difference was observed among emergency cases (Table 2). This finding in the emergency subgroup should be interpreted with caution, as the number of specialist cases was very limited (*n* = 6), resulting in insufficient statistical power.

**Table 1:** Comparison of general characteristics, pregnancy outcome and recurrence

	Specialist	Non-specialist	*P*
*N*	32	90	
Age (y)	33.00 [28.75, 36.25]	30.00 [28.00, 35.00]	0.191
Gravidity	0.00 [0.00, 1.00]	0.00 [0.00, 1.00]	0.861
Parity	0.00 [0.00, 1.00]	0.00 [0.00, 1.00]	0.772
Dysmenorrhea (%)	26 (81.2)	59 (65.6)	0.151
VAS	5.00 [2.00, 6.25]	3.00 [0.00, 6.00]	0.233
Preoperative CA125 (IU/ml)	52.40 [31.98, 112.50]	70.80 [39.42, 124.03]	0.323
Preoperative AMH (ng/ml)	2.77 [2.25, 3.08]	3.23 [1.78, 3.84]	0.710
Elective surgery n (%)	26 (81.2)	41 (45.6)	0.001^*^
Max diameter of OMA by US (cm)	6.80 [5.18, 8.38]	6.05 [5.00, 8.23]	0.253
Intraoperative information			
Location n (%)			0.355
Unilateral	24 (75.0)	76 (84.4)	
Bilateral	8 (25.0)	14 (15.6)	
rAFS score	36.00 [32.00, 76.00]	56.00 [30.00, 76.00]	0.930
Adhesion rate n (%)	22 (88.0)	78 (92.9)	0.718
Obliteration of cul-de-sac n (%)			0.543
Partial	7 (21.9)	20 (22.2)	
Complete	10 (31.2)	37 (41.1)	
Deep infiltrating endometriosis n (%)	8 (25.0)	21 (23.3)	1.000
Adenomyosis n (%)	5 (15.6)	5 (5.6)	0.159
Hemostasis n (%)			
Electrocoagulation	23 (95.8)	67 (79.8)	0.121
Suture	5 (15.6)	10 (11.1)	0.723
Surgery time (min)	55.00 [40.00, 60.00]	60.00 [40.00, 80.00]	0.333
Blood loss (ml)	30.00 [10.00, 50.00]	45.00 [20.00, 50.00]	0.321
Follow up			
Follow-up method n (%)			0.208
By clinic	18 (56.3)	39 (43.3)	
By phone	14 (43.8)	51 (56.7)	
Postoperative AMH (ng/ml)	1.25 [0.53, 2.03]	1.47 [0.71, 3.42]	0.503
Postoperative hormonal therapy n (%)	24 (75.0)	60 (66.7)	0.514
Treatment duration (mo)	5.00 [1.00, 19.00]	3.00 [1.50, 7.25]	0.284
Long-term treatment rate (> 1 year) n (%)	12 (38.7)	18 (21.7)	0.110
Prepare for pregnancy n (%)	9 (28.1)	32 (35.6)	0.625
Clinical pregnancy rate n (%)	7 (77.8)	27 (84.4)	1.000
Type of fertilization n (%)			1.000
Natural conception	6 (85.7)	22 (81.6)	
Assisted Reproductive Technology	1 (14.3)	5 (18.5)	
Recurrence n (%)			0.264
Cyst recurrence	4 (12.5)	26 (28.9)	
Pain recurrence	0 (0.0)	1 (1.1)	
Both	1 (3.1)	3 (3.3)	
Short-term recurrence (< 6 mo) n (%)	0 (0.0)	9 (30.0)	0.385
Five-years cumulative recurrence rate (%)	26.9	34.5	0.033^*, a^
Latency of recurrence-free period (mo)	48.00 [16.00, 60.00]	12.00 [5.00, 30.00]	0.093

^*^ *P* < 0.05

※ *P* value from log-rank test comparing cumulative recurrence-free survival over time

**Table 2:** Recurrence rate by sub-groups of timing of surgery

	Specialist Recurrence/total (%)	Non-specialist Recurrence/total (%)	*P*
Follow-up cohort (*n* = 122)	5/32 (15.6)	30/90 (33.3)	0.057
Emergency surgery (*n* = 55)	1/6 (16.7)	13/49 (26.5)	0.978
Elective surgery (*n* = 67)	4/26 (15.4)	17/41 (41.5)	0.025^*^

**Fig. 3:**
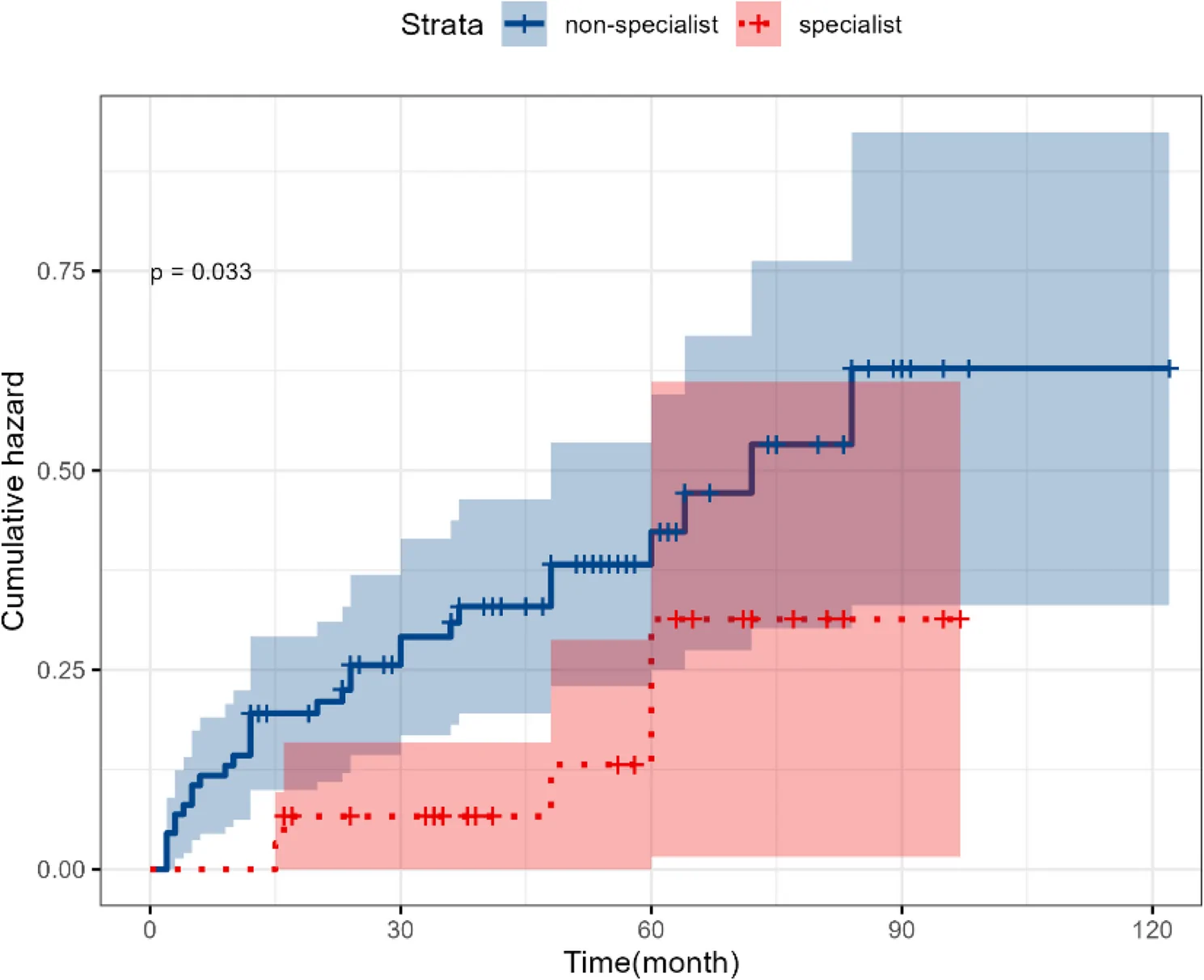
Specialist group is associated with lower cumulative hazard over time compared to non-specialist group

Kaplan–Meier derived cumulative hazard estimates demonstrated a significantly lower cumulative hazard in the specialist group compared to the non-specialist group (*p* = 0.033) (Fig. 3). In multivariable Cox regression, specialist surgery remained associated with a lower hazard of recurrence (adjusted HR 0.378, 95% CI 0.140–1.021), although the association did not reach conventional statistical significance (*p* = 0.055). None of the other covariates were significantly associated with recurrence (Table 3).

**Table 3:** Multivariable Cox proportional hazards regression analysis of factors associated with recurrence

Variable	HR	95% CI	*P*
Specialist surgery vs. non-specialist	0.378	0.140–1.021	0.055
Elective surgery vs. emergency	1.521	0.738–3.134	0.256
Max OMA diameter, per cm	0.983	0.862–1.120	0.793
rASRM score, per point	1.002	0.989–1.014	0.801
Treatment duration, per month	0.992	0.968–1.016	0.496

With regard to ovarian reserve and reproductive outcome, preoperative AMH was available in 24 patients, and postoperative AMH was available in 30 patients. Preoperative and postoperative AMH were comparable. There were no statistically significant differences between the groups in clinical pregnancy rates and in the type of fertilization method used.

## Discussion

Our findings suggest a potential influence of surgical expertise on long-term outcomes in patients with ruptured OMAs. Patients operated on by endometriosis specialists had a lower five-year cumulative recurrence risk compared to those treated by non-specialists (26.9% vs. 34.5%), with a significant difference observed in Kaplan–Meier analysis (log-rank *p* = 0.033). After adjustment for surgery timing, OMA size, rASRM score, and treatment duration, specialist surgery was associated with a lower risk of recurrence (HR 0.378, 95% CI 0.140–1.021), although this did not reach conventional statistical significance (*p* = 0.055), suggesting a consistent trend.

Spontaneous OMA rupture is often reported during the perimenstrual period, when hormonal fluctuations and increased intracystic pressure weaken the cyst wall. The leakage of cystic contents can trigger chemical peritonitis and severe inflammation, potentially leading to tissue edema and pelvic adhesions that complicate subsequent surgery. In addition, given the potential impact of cystectomy on fertility, the optimal timing of surgery remains a matter of ongoing debate. In this context, our findings suggest that regardless of the timing of surgery, surgical expertise may be associated with recurrence risk and long-term outcomes.

Laparoscopic cystectomy for ovarian endometrioma requires advanced surgical skills, including appropriate tissue handling, accurate identification of the cleavage plane, and careful hemostasis to minimize damage to the ovarian cortex. Such meticulous technique may help preserve ovarian anatomy and function, and may contribute to reducing recurrence risk [7, 8]. Histological evidence has suggested an inverse relationship between surgical expertise and inadvertent removal of healthy ovarian tissue [7], which likely reflects the specialists’ heightened awareness and surgical habits. The observed benefit of specialists in our study may therefore reflect not only technical proficiency, but also differences in surgical judgment, experience with endometriosis-specific procedures, and postoperative management.

It is widely recognized that ovarian reserve, represented by the serum AMH levels, declined after surgery, with partial recovery reported one year post-surgery [9]. Factors contributing to this decline include loss of ovarian cortex, use of hemostasis techniques, vascular injury, and postoperative inflammation or edema [10]. Among these, surgical technique is pivotal. While bipolar electrocoagulation has been associated with greater ovarian damage than suturing in some studies [11], other reports show no significant impact based on hemostasis technique [12]. We observed no intergroup difference in the choice of hemostasis method, suggesting both surgical teams took comparable care in fertility preservation.

Peritoneal lavage may represent an additional, yet under-recognized, factor in this context. A limited number of studies have discussed its potential role in endometriosis management [13, 14]. However, direct clinical evidence linking intraoperative peritoneal lavage to reduced recurrence risk remains scarce. Given the retrospective nature of our study, we were unable to reliably determine whether peritoneal lavage was consistently performed. Therefore, its potential influence on recurrence risk warrants further investigation.

In the present study, postoperative treatment rates and duration were numerically higher in the specialist group compared with the non-specialist group; however, these differences did not reach statistical significance. Similarly, in Cox regression analysis, treatment duration was not significantly associated with recurrence risk (HR 0.992), suggesting no clear effect within our dataset.

Nevertheless, existing evidence has consistently suggested that appropriate postoperative hormonal therapy plays an important role in reducing recurrence risk. Previous studies have reported recurrence risk approaching 50% at five years and 27% for 2 years in the absence of long-term postoperative therapy [15, 16]. Appropriate postoperative medical therapy could reduce recurrence risk, with rates dropping to as low as 6% at five years post-surgery [16–18]. In this context, the numerically higher rates and longer duration of postoperative treatment observed in the specialist group may reflect differences in postoperative management practices, including treatment selection, follow-up, and patient counseling. Although our results do not establish a causal relationship, these findings highlight the potential importance of structured postoperative management and specialized training in endometriosis care.

We also observed a higher proportion of elective surgeries in the specialist group compared with the non-specialist group (81.2% vs. 45.6%, *P* = 0.001). The optimal timing of surgery following suspected OMA rupture remains controversial. While early intervention within 72 h has been associated with improved fertility outcomes [19], emergency surgeries are often performed by on-call generalists lacking endometriosis-specific training, which may partly explain the higher proportion of emergency cases in the non-specialist group. Conversely, patients whose symptoms stabilize after initial presentation may defer surgery and subsequently seek care from specialist surgeons, which may be associated with more favorable long-term outcomes. This imbalance in surgical timing introduces the possibility of selection bias. However, in our multivariable Cox regression analysis, surgical timing (emergency vs. elective) was not significantly associated with recurrence, suggesting that the observed benefit of specialist surgery cannot be solely explained by this factor.

From a clinical perspective, an alternative management strategy—such as initial drainage, peritoneal lavage, and medical therapy followed by delayed definitive surgery—has been proposed to allow resolution of the inflammatory milieu and potentially reduce ovarian damage. In our center, however, cystectomy is often performed during the same procedure in emergency settings when an ovarian cyst is identified. This approach may reduce the need for subsequent surgical interventions and may be associated with improved patient acceptance of a single-stage treatment strategy.

Taken together, these findings highlight that decisions regarding the timing of surgery should consider not only the patient’s clinical condition, but also the availability of specialized surgical expertise and the potential risks of operating in an inflammatory environment. As the classification of emergency versus elective surgery in our study reflects real-world clinical decision-making rather than a predefined protocol, these results should be interpreted with caution.

This study has several limitations. First, the retrospective design may introduce inherent biases, including potential selection bias related to surgical timing, as well as incomplete control of confounding factors. Although multivariable Cox regression analysis was performed, the association between specialist surgery and recurrence did not reach conventional statistical significance, suggesting that the observed findings should be interpreted with caution and require further validation in larger cohorts. Second, some follow-up data were obtained via telephone interviews, which may have introduced recall bias. In addition, multiple surgeons were involved, and inter-surgeon variability was not formally assessed. Third, while no significant differences were found in AMH levels or clinical pregnancy rates between groups, this may be due to the limited number of patients who underwent AMH testing or actively attempted conception. As OMA rupture creates an emergency, preoperative ovarian reserve assessment is not always feasible. Previous study suggested a higher natural fertility within the first year after cystectomy when performed by an endometriosis specialist compared to a merely skilled surgeon [6]. Since this study was not specifically designed or sufficiently powered to evaluate fertility, future prospective studies with larger sample size and standardized fertility evaluations are warranted.

## Conclusion

Our study suggests a potential role of surgical expertise in the management of ruptured OMAs. Patients treated by endometriosis specialists showed a lower long-term recurrence risk compared with those managed by non-specialists, with borderline statistical significance after adjustment for potential confounders. These findings highlight the potential importance of structured training and continued professional development in endometriosis surgery and postoperative care, which may contribute to optimizing patient outcomes across different clinical settings.

## Data Availability

The datasets generated and/or analysed during the current study are not publicly available due to individual privacy but are available from the corresponding author on reasonable request.

## Abbreviations

OMA: Ovarian Endometrioma
PUMCH: Peking Union Medical College Hospital
AMH: Anti-Müllerian Hormone
rASRM: Revised American Society of Reproductive Medicine

## References

[1] Jenkins S, Olive DL, Haney AF. Endometriosis: pathogenetic implications of the anatomic distribution. Obstet Gynecol. 1986;67(3):335–8. PubMed PMID: 3945444. 3945444

[2] Pratt JH, Shamblin WR. Spontaneous rupture of endometrial cysts of the ovary presenting as an acute abdominal emergency. Am J Obstet Gynecol. 1970;108(1):56–62. 10.1016/0002-9378(70)90204-8. 5465884

[3] Gu Z, Li X, Dai Y, Shi J, Wu Y, Zhang C, et al. Clinical features of patients with previous spontaneous rupture of ovarian endometrioma operated electively: a case-control study. Reprod Health. 2023;20(1):156. 10.1186/s12978-023-01702-z . PubMed PMID: 37865796; PubMed Central PMCID: PMC10589996. 37865796 PMC10589996

[4] Becker CM, Bokor A, Heikinheimo O, Horne A, Jansen F, Kiesel L, et al. ESHRE guideline: endometriosis. Hum Reprod Open. 2022;2022(2):hoac009. 10.1093/hropen/hoac009. 35350465 PMC8951218

[5] Gu Z, Li X, Shi J, Wu Y, Zhang J, Zhang C, et al. The Development of Predictive Nomogram of Recurrence for Patients With Endometrioma After Cystectomy Who Were Younger Than 45 Years Old and Received Postoperative Therapy. Front Med. 2022;9:872481. 10.3389/fmed.2022.872481. PMC9218256 35755050

[6] Gizzo S, Conte L, Di Gangi S, Leggieri C, Quaranta M, Noventa M, et al. Could surgeon’s expertise resolve the debate about surgery effectiveness in treatment of endometriosis-related infertility? Arch Gynecol Obstet. 2015;292(1):217–23. 10.1007/s00404-014-3591-z. 25524537

[7] Muzii L, Marana R, Angioli R, Bianchi A, Cucinella G, Vignali M, et al. Histologic analysis of specimens from laparoscopic endometrioma excision performed by different surgeons: does the surgeon matter? Fertil Steril. 2011;95(6):2116–9. 10.1016/j.fertnstert.2011.02.034. 21411079

[8] Saridogan E. Role of general gynaecologists in the prevention of infertility. Best Pract Res Clin Obstet Gynecol. 2019;59:132–6. 10.1016/j.bpobgyn.2019.01.014. 30837119

[9] Baraki D, Richards EG, Falcone T. Treatment of endometriomas: Surgical approaches and the impact on ovarian reserve, recurrence, and spontaneous pregnancy. Best Pract Res Clin Obstet Gynecol. 2024;92:102449. 10.1016/j.bpobgyn.2023.102449. 38160479

[10] Carrillo L, Seidman DS, Cittadini E, Meirow D. The role of fertility preservation in patients with endometriosis. J Assist Reprod Genet. 2016;33(3):317–23. 10.1007/s10815-016-0646-z. 26768141 PMC4785156

[11] Ata B, Turkgeldi E, Seyhan A, Urman B. Effect of Hemostatic Method on Ovarian Reserve Following Laparoscopic Endometrioma Excision; Comparison of Suture, Hemostatic Sealant, and Bipolar Dessication. A Systematic Review and Meta-Analysis. J Minim Invasive Gynecol. 2015;22(3):363–72. 10.1016/j.jmig.2014.12.168. 25573183

[12] Takashima A, Takeshita N, Otaka K, Kinoshita T. Effects of bipolar electrocoagulation versus suture after laparoscopic excision of ovarian endometrioma on the ovarian reserve and outcome of in vitro fertilization. J Obstet Gynaecol Res. 2013;39(7):1246–52. 10.1111/jog.12056 . PubMed PMID: 23803008. 23803008

[13] Smith LP, Williams CD, Doyle JO, ’Brien, Closshey WB, Brix WK, Pastore LM. Effect of endometrioma cyst fluid exposure on peritoneal adhesion formation in a rabbit model. Fertil Steril. 2007;87(5):1173–9. 10.1016/j.fertnstert.2006.08.104. 17258215

[14] Grammatis AL, Lazaridis A, Becker CM, Saridogan E. Surgical techniques for ovarian endometriosis. Obstetrician Gynaecologist. 2024;26(4):197–208. 10.1111/tog.12947.

[15] Veth VB, Keukens A, Reijs A, Bongers MY, Mijatovic V, Coppus SFPJ, et al. Recurrence after surgery for endometrioma: a systematic review and meta-analyses. Fertil Steril. 2024;122(6):1079–93. 10.1016/j.fertnstert.2024.07.033 . PubMed PMID: 39098538. 39098538

[16] Lee DY, Bae DS, Yoon BK, Choi D. Post-operative cyclic oral contraceptive use after gonadotrophin-releasing hormone agonist treatment effectively prevents endometrioma recurrence. Hum Reprod. 2010;25(12):3050–4. 10.1093/humrep/deq279 . PubMed PMID: 20937741. 20937741

[17] Zakhari A, Delpero E, McKeown S, Tomlinson G, Bougie O, Murji A. Endometriosis recurrence following post-operative hormonal suppression: a systematic review and meta-analysis. Hum Reprod Update. 2021;27(1):96–107. 10.1093/humupd/dmaa033. 33020832 PMC7781224

[18] Chiu CC, Hsu TF, Jiang LY, Chan IS, Shih YC, Chang YH, et al. Maintenance Therapy for Preventing Endometrioma Recurrence after Endometriosis Resection Surgery – A Systematic Review and Network Meta-analysis. J Minim Invasive Gynecol. 2022;29(5):602–12. .024 PubMed PMID: 35123042. 35123042 10.1016/j.jmig.2021.11.024

[19] Huang YH, Hsieh CL, Shiau CS, Lo LM, Liou JD, Chang MY. Suitable timing of surgical intervention for ruptured ovarian endometrioma. Taiwan J Obstet Gynecol. 2014;53(2):220–3. 10.1016/j.tjog.2014.04.018. 25017271

